# Which Is Stronger? A Continuing Battle Between Cry Toxins and Insects

**DOI:** 10.3389/fmicb.2021.665101

**Published:** 2021-06-01

**Authors:** Lu Liu, Zhou Li, Xing Luo, Xia Zhang, Shan-Ho Chou, Jieping Wang, Jin He

**Affiliations:** ^1^State Key Laboratory of Agricultural Microbiology, College of Life Science and Technology, Huazhong Agricultural University, Wuhan, China; ^2^Department of Molecular Biology, Qingdao Vland Biotech Inc., Qingdao, China; ^3^Agricultural Bioresources Institute, Fujian Academy of Agricultural Sciences, Fuzhou, China

**Keywords:** *Bacillus thuringiensis*, Cry toxin, receptor, insecticidal mechanism, resistance mechanism

## Abstract

In this article, we review the latest works on the insecticidal mechanisms of *Bacillus thuringiensis* Cry toxins and the resistance mechanisms of insects against Cry toxins. Currently, there are two models of insecticidal mechanisms for Cry toxins, namely, the sequential binding model and the signaling pathway model. In the sequential binding model, Cry toxins are activated to bind to their cognate receptors in the mid-intestinal epithelial cell membrane, such as the glycophosphatidylinositol (GPI)-anchored aminopeptidases-N (APNs), alkaline phosphatases (ALPs), cadherins, and ABC transporters, to form pores that elicit cell lysis, while in the signaling pathway model, the activated Cry toxins first bind to the cadherin receptor, triggering an extensive cell signaling cascade to induce cell apoptosis. However, these two models cannot seem to fully describe the complexity of the insecticidal process of Cry toxins, and new models are required. Regarding the resistance mechanism against Cry toxins, the main method insects employed is to reduce the effective binding of Cry toxins to their cognate cell membrane receptors by gene mutations, or to reduce the expression levels of the corresponding receptors by trans-regulation. Moreover, the epigenetic mechanisms, host intestinal microbiota, and detoxification enzymes also play significant roles in the insects’ resistance against Cry toxins. Today, high-throughput sequencing technologies like transcriptomics, proteomics, and metagenomics are powerful weapons for studying the insecticidal mechanisms of Cry toxins and the resistance mechanisms of insects. We believe that this review shall shed some light on the interactions between Cry toxins and insects, which can further facilitate the development and utilization of Cry toxins.

## Introduction

*Bacillus thuringiensis* (Bt) is a spore-producing Gram-positive bacterium (Li Z. et al., 2020). Its distinguishing feature is the formation of abundant parasporal crystals during sporulation (Wang et al., 2013a,b), which typically comprise various crystal toxins (Cry toxins) and cytolytic toxins (Cyt toxins). Owing to their specificity and diversity, such toxins were also found to be environmentally friendly agents to kill Lepidoptera, Diptera, Coleoptera, and other target insects (Fu et al., 2018). Therefore, Bt preparations are currently the most productive and widely used microbial insecticides in agriculture and forestry industries (Mendoza-Almanza et al., 2020).

In addition to Cry and Cyt (collectively known as δ endotoxin), the toxins secreted by Bt also include α-exotoxin (such as phospholipase C), β -exotoxin (such as thuringiensin), secreted insecticidal proteins (Sip), vegetable insecticidal protein (Vip), and other exotoxins (Xiao and Wu, 2019). Among them, Cry, Cyt, Sip, and Vip are the main Bt insecticidal proteins. To evaluate the diversity and function of Bt insecticidal proteins, Crickmore et al. (2020) have, based on the sequence and structures, renamed and compiled them into a new database, including those not previously included (Crickmore et al., 1998). According to the latest naming system, Bt insecticidal proteins are now classified into 16 categories, the largest among which are still the Cry toxins; however, this category of toxins currently includes only those with the classic three-domain Cry toxins. In fact, other Cry toxins by the original nomenclature system are now divided into novel groups with new names, such as Tpp35Aa (previously Cry35Aa), Tpp1Aa (previously BinA), Mpp51Aa (previously Cry51Aa), Mpp2Aa (previously Mtx2), Gpp34Aa (previously Cry34), and App6Aa (previously Cry6Aa). We will thus, in this article, describe only those three-domain Cry toxins that are the most popular and well-studied. Investigation of the structures and functions of Cry toxins is rather challengeable and interesting, since most Cry toxins act by recognizing specific cell membrane receptors such as cadherins, glycophosphatidylinositol (GPI)-anchored aminopeptidases-N (APNs), alkaline phosphatases (ALPs), and ABC transporters. Here we shall focus only on the description of Cry toxins and discuss their insecticidal mechanisms as well as the resistance mechanisms of different insects against these Cry toxins. We believe that this manuscript shall set a basis for the further research, development, and utilization in this important and practical field.

## The Main Structure of Cry Toxin

**FIGURE 1:**
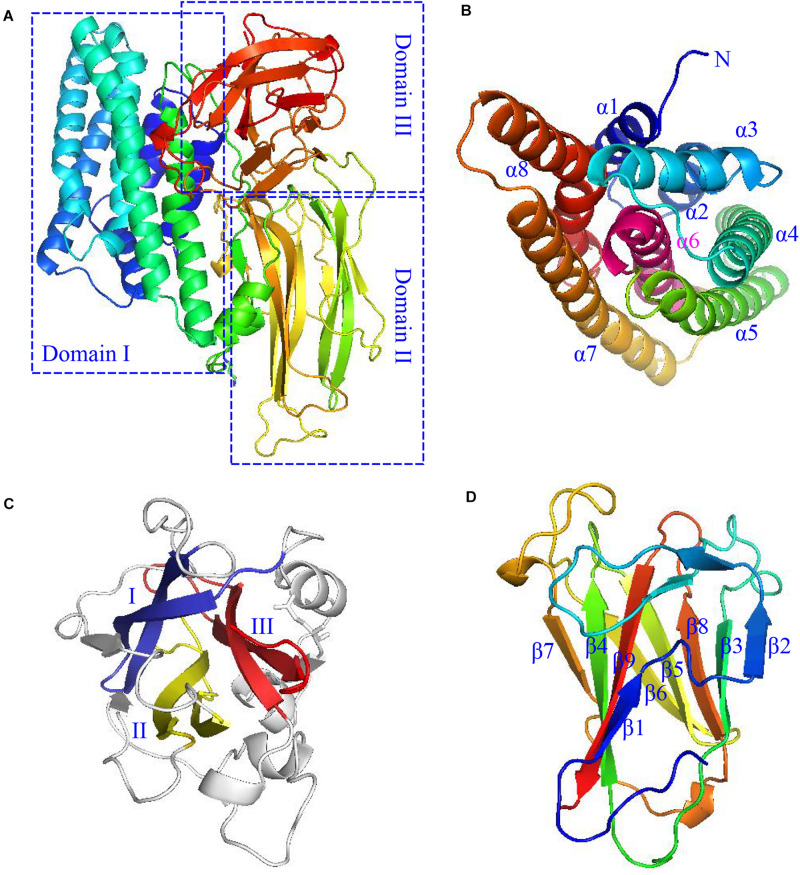
The typical structure of a Cry toxin (Cry1Ac with the PDB code of 4ARX) produced by Btk HD-73. **(A)** The three domains are each boxed in the dotted blue line, and enlarged in different orientations for the better viewing of their unique domain structures. **(B)** The domain I structure, which adopts mostly helical structures, is drawn in rainbow color and labeled from α1 to α8 (note that α8 is highly bent), except that of the unique middle α6 helix, which is colored and labeled in magenta. **(C)** The domain II structure. The three anti-parallel β-sheets are arranged in circular mode and drawn in blue, yellow, and red, respectively. The loop regions and coil residues are drawn in gray. **(D)** The Domain III structure, which comprises two anti-parallel β-sheets comprising the β1–β9–β4–β7 and β2–β3–β8–β5–β6 strands, respectively, are drawn face to face in rainbow color.

Cry protoxins from parasporal crystals comprise two main types according to their molecular weights. One of them is the larger ones with molecular weights of about 130 kDa, such as Cry1Aa; the other one is the smaller ones with molecular weights of approximately 65–70 kDa, such as Cry11Aa. Larger protoxins are processed by the insect mid-intestinal protease at both the C- and N-termini (Jurat-Fuentes et al., 2021), while smaller protoxins are truncated only at the N-terminus. Yet, both types of protoxins form active Cry toxins of approximately 60∼70 kDa eventually. They are usually consisted of three conserved domains, with each one exhibiting a specific function (Pardo-López et al., 2013). Taking the active Cry1Ac (PDB: 4ARX) as an example (Figure 1), domain I is located at the N-terminus of the protein and consisted of an eight-α-helical bundle normally associated with the mid-intestinal epithelial cell membrane insertion and pore formation (Bravo et al., 2007; Palma et al., 2014). Domain I is rather unusual, as it contains a conserved hydrophobic helix α6 in the middle of the helix bundle and is surrounded by six neighboring helices (Figure 1B). The middle domain II is also uncommon, as it is composed of three antiparallel β-sheets arranged in a circular mode to form a hydrophobic core with some highly variable and exposed loop regions (Figure 1C), which are often suggested to confer the binding specificity of the Cry toxin with the mid-intestinal epithelial cell membrane receptors of target insects (Bravo et al., 2007; Evdokimov et al., 2014). Domain III, in contrast, forms a regular β-sandwich structure composed of two antiparallel β-sheets (Figure 1D; Palma et al., 2014; Xu et al., 2014), which typically participates in the specific binding with receptors such as N-acetylgalactosamine in the APN (Bel et al., 2020), as well as in forming pores on the cell membranes (Xu et al., 2014; Mendoza-Almanza et al., 2020). Besides, other domains in the protoxins may also participate in the stabilization of the various unique Cry toxin structures, in their selective dissolution and specific receptor recognition (Palma et al., 2014).

## The Insecticidal Mechanisms of Cry Toxins

Although the insecticidal mechanisms of Cry toxins are not yet fully understood, based on existing information, two models have been proposed, namely, the sequential binding model (Jenkins et al., 2000; Gómez et al., 2002) and the signaling pathway model (Zhang et al., 2005, 2006; Vachon et al., 2012).

### The Sequential Binding Model

**FIGURE 2:**
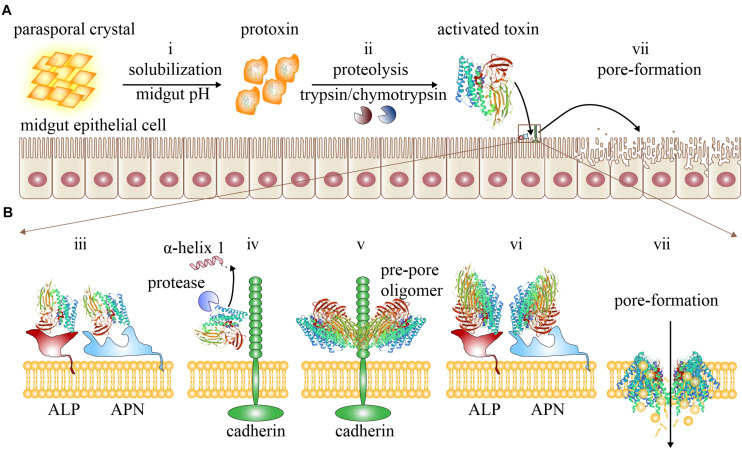
The sequential binding model. **(A)** The activation process of Cry toxins and the pore formation in the mid-intestinal epithelial cells: (i) The parasporal crystals are first dissolved and released into insect mid-intestinal fluid to form protoxins. (ii) Protoxins are then hydrolyzed by proteases to form active monomeric Cry toxins. **(B)** The active Cry toxins can bind to mid-intestinal epithelial cell membrane receptors sequentially to initiate pore formation: (iii) The monomeric Cry toxins first bind to the cell membrane receptors ALP and APN reversibly with low affinity. (iv) The monomeric Cry toxins enriched around the membrane region then bind to the cadherin receptors irreversibly with higher affinity, facilitating the proteolysis of α-helix 1 at the N-terminal of domain I. (v) The cleaved monomeric Cry toxins can now bind with cadherins to form pre-pore oligomers. (vi) After forming the pre-pore oligomer, the Cry toxins can bind to ALP and APN with a higher affinity. (vii) The oligomers are now driven to insert into the mid-intestinal epithelial cell membrane, causing perforation and cell lysis.

As described, a rough but generally accepted insecticidal mechanism of Cry toxins is the sequential binding model or the classic mode of action (Khorramnejad et al., 2020). The sequential binding model comprises a complicated multistep process (Figure 2), emphasizing on the specific binding of Cry toxins to a variety of receptors (Fu et al., 2018). The primary feature of this model is that when Cry toxins bind specifically to the various mid-intestinal epithelial cell membrane receptors in a sequential manner (Figure 2B), these toxins become more mature and form oligomers that are subsequently inserted into the cell membrane, causing perforations and osmotic imbalance and ultimately leading to the cell lysis and insect death (Palma et al., 2014; Melo et al., 2016).

In this model, the parasporal crystals are first dissolved in the mid-intestinal fluid to release the protoxins when ingested by insects (Figure 2Ai). Under appropriate pH conditions, the protoxins are cleaved by the digestive proteases such as trypsin and chymotrypsin (Figure 2Aii). After the removal of the N- and/or C-terminals, these monomeric Cry toxins become active and can pass through the peritrophic matrix to reach the apical brush border membrane of the insect mid-intestine (Mendoza-Almanza et al., 2020). Next, sequential binding to diverse receptors occurs and is likely the key to insecticidal activity (Figure 2B). Although the exact mechanism remains unclear, based on the available experimental evidences, some researchers propose the following processes: First, the exposed loop of domain II in monomeric Cry toxin can recognize and bind specifically and reversibly with the GPI-anchored APN and ALP receptors with a moderately strong affinity (with a Kd of 750 nM) (Figure 2Biii; Gómez et al., 2007; Vachon et al., 2012). After Cry toxins are localized and enriched onto the membrane (Xu et al., 2014), they can bind irreversibly to the ectodomain of the cadherin receptor with a stronger affinity (with a Kd of 1 nM, Figure 2Biv; Gómez et al., 2007; Vachon et al., 2012). Following binding, the Cry toxins are induced to undergo conformational changes (Vachon et al., 2012; Palma et al., 2014), which facilitate the proteolytic cleavage of the α-helix 1 from the N-terminal of domain I. The mature Cry toxins now leave the cadherin receptor to form pre-pore oligomers (Figure 2Bv), which then bind again to the APN and ALP receptors with greater affinity compared with monomeric Cry (Figure 2Bvi; Bravo et al., 2004; 2007) and drive their insertion into the cell membrane (Bravo et al., 2004, 2007; Palma et al., 2014) to form a crucial transmembrane ion channel (Figure 2Bvii). This process disrupts the integrity of the cell membrane and allows different types of ions to pass freely, which can significantly perturb the cellular physiological and osmotic balance, ultimately leading to cell lysis. In addition, the pores also allow intestinal contents, such as bacteria, to leak into the hemocoel (Fu et al., 2018), which can cause sepsis and trigger insect death.

In the sequential binding model, only certain ABC transporters are believed to be the functional integral membrane receptors of Cry toxins, such as ABCC2 (Ocelotl et al., 2017) and ABCB1 (Sato et al., 2019); they are versatile and exhibit multiple functions, including the ability to export toxic molecules from cytosol (Gahan et al., 2010). They can also involve in forming the oligomeric complex for insertion into the cell membrane. Since the ABC transporter was discovered as one of the Cry toxin receptors later, it is currently unclear how they bind to Cry toxins, whether they interact with other gene products or exhibit any other unknown functions for Cry toxicity. Additionally, other intracellular proteins, such as actin, flotillin, prohibitin, and V-ATPase, are also potentially involved in the binding of Cry toxins, but the role of these proteins remains unknown (Palma et al., 2014). The binding affinity of Cry toxins to the cognate receptors is determined by the Cry toxin domains II and III, the species and numbers of receptors in the binding sites of the insect mid-intestinal epithelial cell membrane (Pigott and Ellar, 2007), and the mid-intestinal pH value (Chattopadhyay and Banerjee, 2018).

According to the sequential binding model, the proteolytic cleavage of α-helix 1 and pre-pore formation are the prerequisites for the function of Cry toxins (Jiménez-Juárez et al., 2008). However, there are also exceptions, as previous studies have shown that pore formation can still occur without forming pre-pores when a certain Cry toxin retains the intact α-helix 1 but with other regions being cleaved (Melo et al., 2016). This indicates that the proteolytic cleavage of α-helix 1 and pre-pore oligomers are possibly not absolutely required for the pore formation in the sequential binding model, indicating that there may exist other pathways for the insecticidal mechanisms of Cry toxins.

### The Signaling Pathway Model

**FIGURE 3:**
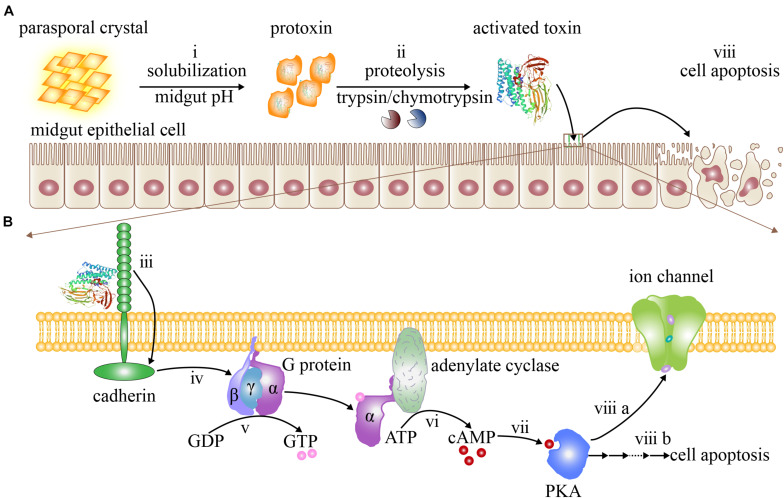
The signaling pathway model. **(A)** The activation process of Cry toxins and cell apoptosis in mid-intestinal epithelial cells. (i) Parasporal crystals are dissolved and released into insect intestinal fluid after ingestion by insects. (ii) Protoxins are hydrolyzed and activated to release active Cry toxins. **(B)** A series of cell signaling cascade pathways are activated by the binding of Cry toxins with the cadherin receptors, leading to cell apoptosis. (iii) Active Cry toxins bind to cadherin receptors. (iv) A series of Mg^2+^-dependent downstream cell signaling cascades are triggered. (v) G proteins are activated to synthesize GTP. (vi) Adenylate cyclases are now activated to synthesize cAMP. (VII) PKAs are activated after binding with cAMP. (VIII) Activated PKAs then destabilize ion channels in the mid-intestinal epithelial cell membrane (a), or trigger further downstream cell signaling cascades, leading to cell apoptosis (b).

Like the activation pathway of the sequential binding model (Melo et al., 2016), the signaling pathway model (also named alternate mode of action) also comprises leading steps such as parasporal crystal dissolution and protoxin formation (Figure 3Ai), protoxin proteolysis and generation of active Cry toxin (Figure 3Aii), and receptor recognition and binding (Figure 3B). However, the subsequent steps of this model are completely different to the sequential binding model in that cell death is caused not by the insertion of Cry toxins into the mid-intestinal epithelial cell membrane for pore formation (Figure 2Avii) but by the cellular apoptosis mediated by the cadherin receptors (Figure 3; Palma et al., 2014). According to this hypothesis, once Cry toxins recognize and specifically bind to cadherin receptors (Figure 3Biii), they will provoke several Mg^2+^-dependent cell signaling cascades (Figure 3Biv), such as the activation of G proteins (Figure 3Bv) and adenylate cyclase to increase the level of the intracellular second messenger molecule cAMP (Figure 3Bvi), which will further activate protein kinases A (PKAs) (Figure 3Bvii) to trigger a series of downstream cell signaling cascade pathways (Figure 3Bviii), finally leading to the disruption of ion channels (Figure 3Bviiia) and cytoskeletons (Pigott and Ellar, 2007; Xu et al., 2014), as well as acceleration of cell apoptosis (Figure 3Aviii), ultimately causing insect death (Zhang et al., 2006).

The signaling pathway model does not involve interactions with other protein receptors such as GPI-anchored APNs and ALPs, or the formation of pre-pore oligomers and pores, thus substantially simplifying the long process of interaction between Cry toxins and cell membrane receptors (Zhang et al., 2005). This model can also explain why some insects can still be killed by Cry toxins when no receptor other than the cadherin is present.

### Other Possible Insecticidal Mechanisms

Although these two insecticidal models are different from each other to certain extents, some scholars have pointed out that they may work together (Lacey et al., 2015). For example, the Cry toxins undergoing mutations that affect oligomerization or pore formation can result in insufficient binding to cadherins, which reduces the insecticidal activity of Cry toxins to produce only partial resistance, indicating that the signaling pathway model may be affected by the sequential binding of Cry toxins with the receptors (Xu et al., 2014; Melo et al., 2016). In addition, although Cry1AMod toxin without the N-terminus (including α-helix 1) of domain I is unable to bind to cadherin, it can still kill target insects. This indicates that other than the two mechanisms mentioned above, there may exist other action modes for the Cry toxins (Pardo-López et al., 2013).

### The Death Process of Insects

In reality, the killing process of Cry toxins toward insects is extremely complicated, which starts from cell damage (Heckel, 2020). On the one hand, Cry toxins induce damage of certain insect cells, such as mid-intestinal epithelial cells, which cause the interruption of insect feeding and affect the final survival of insects; on the other hand, during the epithelial cell damage, the Cry toxins lead to imbalance of the intestinal microbiota homeostasis. Then, the dysregulated intestinal microbiota and Cry toxin join to stimulate the excessive intestinal immune response (Mason et al., 2011) and aggravate the damage to host tissues such as the peritrophic matrix, which causes some intestinal opportunistic pathogens or pathogens to enter into the hemocoel, leading to their rapid proliferation which significantly increases the total bacterial load in the hemolymph, causing host sepsis and accelerating the insect death (Caccia et al., 2016; Li et al., 2021). In addition, others claim that after cell damage, water molecules can enter the cell more easily through aquaporin to combat the ion imbalance caused by the Cry toxins; yet, excessive water entry will induce the cells to swell, leading to their death (Endo et al., 2017).

Although these studies have deepened our understanding on the insecticidal mechanism of Cry toxins, they are still not comprehensive enough. Actually, insect death may be a concurrent comprehensive effect of multiple mechanisms. We believe that with the improvement of research techniques and powerful methods, such as transcriptomics, proteomics, metabonomics, and epigenomics, more secrets regarding the insecticidal mechanism of Cry toxins will be deduced soon.

## The Resistance Mechanisms of Insects Against Cry Toxins

With the widespread use of Bt preparations and Bt-transgenic crops, an increasing number of insects are found to develop resistances against Cry toxins. This alarming situation has thus attracted widespread attention and discussion. Theoretically, insect resistances can occur when any mechanistic step of the insecticidal process is disrupted (Xiao and Wu, 2019), including chiefly the diminution of the “toxin-receptor” binding affinity due to the mutations of receptors’ genes (Chattopadhyay and Banerjee, 2018). In addition, the epigenetic mechanisms, the reduction of the receptors’ expression levels utilizing a trans-regulation mechanism, the presence of intestinal microbiota, or the production of detoxification enzymes may all contribute to the development of insect resistances. In fact, some insects often use multiple resistance strategies simultaneously to cunningly avoid the harm by the Cry toxins. In the following section, we describe the potential resistance mechanisms against Cry toxins in different insects.

### Receptor Gene Mutations Promote Insect Resistance

In the long-term evolution, insects usually adopt the strategy of receptor gene mutations to weaken the effective binding between receptors and cognate Cry toxins to protect themselves from damage by the Bt and Cry toxins (Bel et al., 2020).

The diamondback moth (*Plutella xylostella*) NO-QA strain obtained by artificial selection exhibits extremely high resistance to Cry1Aa, Cry1Ab, Cry1Ac, and Cry1F because the gene encoding a receptor shared by these four Cry toxins has undergone an autosomal recessive mutation, which significantly reduces the binding ability of the toxins to the receptor (Hernández-Martínez et al., 2012). As mentioned above, ABC transporters can facilitate oligomer membrane insertion in late stage of action mode of Cry toxins (Gahan et al., 2010). Thus, the mutations of ABC transporters may disrupt the binding of Cry toxins to BBMVs. Seven different sources of Bt var. *kurstaki* (Btk)-resistant *P. xylostella* strains and NO-QA contain a common polygenic resistant site called *BtR-1* (Baxter et al., 2011; Guo et al., 2015), which is a deletion mutation that occurred in the last transmembrane domain of the *ABCC2* gene that leads to insect resistance. Recent studies have shown that *P. xylostella* requires double mutations of both *ABCC2* and *ABCC3* genes to obtain a certain degree of resistance to Cry1Ac (Liu et al., 2020).

Similarly, cadherin also plays an important role in inducing toxin oligomerization, mediating toxins binding to GPI-anchored receptors in the sequential binding model, or mediating signal transduction in the signaling pathway model. It is because cadherin exhibits so many functions; thus, mutations in the cadherin receptors are considered one of the most ubiquitous resistance mechanisms observed to date. The resistance of pink bollworm (*Pectinophora gossypiella*) to Cry1Ac is related to the mutations of the cadherin receptor (Mohan et al., 2016), with most of them occurring on autosomes, and are recessive (Mohan et al., 2016; Nair et al., 2016). In the resistant population of *P. gossypiella*, there are three alleles encoding the cadherin, among which mutations in the *t2* allele cause changes in eight amino acids linked with the binding of Cry1Ac, which reduces the binding ability with receptor protein. The cell signaling cascade is thus prohibited, leading to insect resistance to Cry toxins. In addition, studies have found that the *ABCA2* gene in *P. gossypiella* undergoes alternative splicing, resulting in the loss of exon 6 and truncation of *ABCA2*. This mutation is highly resistant to Cry2Ab (Mathew et al., 2018).

ABCC2 is a functional receptor of fall armyworm (*Spodoptera frugiperda*) that binds with Cry1Fa, Cry1Ab, and Cry1Ac (Banerjee et al., 2017; Flagel et al., 2018). At present, it is found that all insertion mutations occurring at the same locus of *ABCC2* (Camargo et al., 2017) can cause *S. frugiperda* to develop resistance and cross-resistance to different Cry toxins (Bernardi et al., 2015; Jakka et al., 2015; Monnerat et al., 2015). At the same time, deletions and substitution mutations of two residues in the conserved region of the extracellular loop 4 of ABCC2 receptor can cause *S. frugiperda* to develop resistance to Cry1F (Boaventura et al., 2020; Abdelgaffar et al., 2021).

CaLP (cadherin-like protein) and ABCC2 are genetically related to the resistance of tobacco budworm (*Heliothis virescens*) against Cry1Ac toxin (Bretschneider et al., 2016). The inactivating mutation of *ABCC2* in *H. virescens* is an important reason for the reduced binding of Cry1Ac (Gahan et al., 2010; Baxter et al., 2011). Further studies have shown that ABCC2 is the central receptor of Cry1A, and CaLP is an assisting protein involved in enhancing the toxicity of Cry1A (Bretschneider et al., 2016).

The allele responsible for the Cry2Ab resistance in cabbage looper (*Trichoplusia ni* GLENCry2Ab-BCS) is, however, unrelated with either cadherin, ALP, APN, or ABCC2 receptor. The resistant gene was, in fact, mapped to the genes *ABCA*1 and *ABCA*2 encoding ABC transporters on chromosome 17 (Song et al., 2015). The latest research shows that inactive mutations of the *ABCA*2 gene, but not the *ABCA1* gene in the mid-intestinal epithelial cells, are the main determinants that confer the resistance of *T. ni* to Cry2Ab (Yang et al., 2019).

Yellow fever mosquito (*Aedes aegypti*) has high-frequency mutations in the gene encoding the cadherin receptor, which promotes its resistance against the Bt subsp. *israelensis* (Bti) strain (Bonin et al., 2009). In sweet potato weevil (*Cylas puncticollis*), the mutant site that binds with the three toxins Cry3Bb, Cry3Ca, and Cry7Aa in the BBMV can promote the generation of cross-resistance (Hernández-Martínez et al., 2014). ABCB1 in leaf beetle (*Chrysomela tremulae*) is a functional receptor of Cry3Aa, and a frameshift mutation in its encoding gene can confer resistance to Cry3Aa in *C. tremulae* (Pauchet et al., 2016; Domínguez-Arrizabalaga et al., 2020).

In general, the so-called insect Cry toxin receptor knockout strains do not imply deletion of an entire ABC transporter or cadherin receptor or other receptors, but just lack of a key Cry toxin-binding site, or even a mere 2–5-bp base deletion, which is enough to confer extremely high resistance in insects (Guo et al., 2019; Jin et al., 2021). It is exactly because these small but vital structural changes in the receptors can induce insects to become rather Cry toxin-resistant; thus, it is concluded that these proteins are arguably the most significant functional receptors. This further implies that the research on insect resistance mechanism and the insecticidal mechanisms of Cry toxins are inseparable.

### Epigenetic Mechanisms Promote Insect Resistance

Epigenetic mechanism refers to the environmental stimuli that can be transformed into transgenerational inherited variation without genetic changes (Gómez-Díaz et al., 2012; Laland et al., 2014; Skinner, 2015). Since insects’ adaptation to Bt can occur in a short evolutionary time scale, which is unlikely to be determined only by irreversible genetic changes. Hence, in recent years, more and more studies have pointed out the possibility that epigenetic mechanisms like DNA methylation (Vilcinskas, 2016), histone acetylation modification (Mukherjee et al., 2012), and level changes of microRNAs (miRNAs) (Asgari, 2013; Mukherjee and Vilcinskas, 2014), which are related to insect immunity and development, are involved in the evolution of insect resistance to biological pesticides (Jones et al., 2018).

For example, after the greater wax moth (*Galleria mellonella*) is infected with Bt for 20 generations, the stress and immune defense-related genes in intestine and fat body are transcriptionally reprogrammed, with their expression levels greatly increased, and their immune adaptation to Bt significantly enhanced (Dubovskiy et al., 2016). After further exposing *G. mellonella* larvae to Bt spores and crystals mix for 30 generations, insect strains with enhanced resistance to Bt and Cry toxins could be selected. It is found that the levels of DNA methylation and histone acetylation of this resistant strain are increased, with the expression levels of some conserved miRNAs and their target mRNAs significantly changed, indicating that epigenetic mechanisms mediate the evolution of *G. mellonella* resistance to Bt at pre-transcriptional and posttranscriptional levels (Mukherjee et al., 2017). Another evidence comes from the cotton bollworm (*Helicoverpa armigera*), which, when continuously exposed to Cry1Ac toxin for 12 generations, reveals tolerance to Cry1Ac with enhanced immune status through an epigenetic mechanism from a strong maternal effect, which can be passed to its offspring (Rahman et al., 2011). Besides, the red flour beetle (*Tribolium castaneum*) exhibits an increased survival rate after exposure to Bt in a short time that can be directly transmitted to the first (F1) and second filial (F2) generations, called paternal trans-generational immune priming, which may be regulated by epigenetic mechanisms too (Eggert et al., 2014; Schulz et al., 2019).

It can thus be seen that epigenetic mechanisms seem to play a rather important role in the evolution of insect resistance to Bt or Cry toxins.

### Reduced Expression Levels of Receptors Promote Insect Resistance

The reduced expression levels of receptors can be insufficient for binding Cry toxins to trigger insect resistance, as detected in the following several instances.

The transcriptome and proteome analyses of *A. aegypti*-resistant strain reveal that the downregulation of the cognate receptor expression can effectively inhibit larval death (Tetreau et al., 2012; Després et al., 2014). In the mid-intestinal epithelial cells of *A. aegypti* strain, large decreases in the transcription levels of *ALPs* and *APNs* are found to promote its resistance to Cry11Aa (Lee et al., 2014; Chen et al., 2017), while the resistances of *A. aegypti* to Cry4Aa, Cry4Ba, and Cry11Aa, as well as to Bt strain, are associated with the decrease of the *ALP* transcription levels (Stalinski et al., 2016).

It is also observed that some trans-regulatory mechanisms are responsible for the declining expression levels of several Cry toxin receptors. For example, the mitogen-activated protein kinase (MAPK) signaling cascade can trans-regulate the expression of *ALP* and *ABCC* genes (Peterson et al., 2017), resulting in four *P. xylostella* strains being highly resistant against Cry1Ac (Guo et al., 2015). Under the influence of the *MAP4K4* gene located at the *BtR-1* locus, the ABCC2-3 and ALP expression levels are downregulated, while when the *MAP4K4* gene is knocked out, the expression levels of ALP and ABCC2-3 can be reestablished to restore the insect sensitivity to Cry1Ac. These results reveal that the MAPK signal transduction and trans-regulation of *ALP* and *ABCC* genes are important reasons for *P. xylostella* resistance (Guo et al., 2015).

Similarly, the trans-regulations of APN1 and APN6 are responsible for the *T. ni*’s resistance to Cry1Ac (Baxter et al., 2011; Tiewsiri and Wang, 2011), although the exact mechanism has not been revealed yet. When APN1 is downregulated, APN6 is, as a compensatory mechanism, upregulated, which seems to inhibit the toxicity of Cry1Ac and promotes the insect resistance. The reduced expression of receptors, e.g., aminopeptidase-P like proteins, ALPs, and ABC transporters in rice stem borer (*Chilo suppressalis*), is found to promote its resistance to Cry1C, which is also relevant to the transcription levels of enzymes evolved in the hydrolysis and activation of Cry toxin in the mid-intestine (Chen et al., 2020).

Recently, a new trans-regulation mechanism has also been reported, that is, via microRNA; for example, microRNA 998-3p can downregulate the ABCC2 expression by targeting on *ABCC2* and promote the resistance of lepidopteran insects, including *H. armiera* and *P. xylostella*, to Cry1Ac (Zhu et al., 2020).

### Indigenous Intestinal Microbiota Promotes Insect Resistance

The intestinal microbiota contained in insects varies greatly with insect species, feeding habits, and living environment (Xiao et al., 2017; Li S. et al., 2020). The intestinal microbiota of insects is found to be directly related to the physiological activities of the host, such as nutrient utilization, immune defense and regulation, metabolism, and development. Currently, many studies have revealed that there is a close relationship between intestinal microbiota and insect resistance to Cry toxins (Visweshwar et al., 2015; Wu et al., 2020; Wang et al., 2021).

The intestinal microbiota promotes the resistance of insects to Cry toxins mainly through the following mechanisms: (1) By degrading Cry toxins in the intestinal environment to nullify its toxicity. The indigenous intestinal microbiota of Asian malaria mosquito (*Anopheles stephensi*) promotes the development of resistance through utilizing Bt proteins (including Cry toxins) as nitrogen source only under microaerophilic conditions in line with the natural larval mid-intestine (Patil et al., 2013). The pH in the anterior region of the mid-intestine of pea aphid *Acyrthosiphon pisum* (Harris) is acidic rather than alkaline, which is indispensable for complete toxin solubilization. Cry3Aa thus cannot be completely processed and degraded in its intestine, nor can it bind to the BBMVs, resulting thus in a very low aphicidal activity (Li et al., 2011). (2) By triggering the host mid-intestinal immune response to promote resistance (Emery et al., 2017). Due to its ecological and economic importance, the western honeybee (*Apis mellifera*) is often used to assess the environmental risks of genetically engineered insect-resistant (IRGE) crops (Wang et al., 2015). After *A. mellifera* is fed with Cry1C and Cry2A, its native intestinal microbiota can trigger mid-intestinal immune response, causing upregulation of the encoding genes of antimicrobial peptides apidaecin and hymenoptaecin in the intestinal lumen and hemolymph, which improve the host immune response and promote its resistance to Cry1C and Cry2A (Kwong et al., 2017; Wang et al., 2017). (3) By affecting the aminopeptidase activity and interfering with the binding of Cry toxins to the receptors on BBMVs. After the *H. armigera* intestinal microbes are eliminated by antibiotics, the aminopeptidase activity and the binding of Cry1Ac to receptors on BBMVs are substantially affected, leading to significantly reduced insect mortality (Visweshwar et al., 2015). (4) By decreasing microbiota diversity to favor the host’s defense against Bt infection, which is conserved among different insect species. For example, *A. aegypti* larvae have the lowest intestinal microbiota diversity but exhibit the highest tolerance to Bti (Tetreau et al., 2018). Also, the bacterial abundance of resistant strains of western corn rootworm (*Diabrotica virgifera*) is lower than that of susceptible strains (Paddock et al., 2021). (5) By presenting certain specific intestinal bacteria to help the host to resist Bt infection and Cry toxicity. *Enterococcus mundtii* strains isolated from the Mediterranean flour moth (*Ephestia kuehniella*) larval feces have broad-spectrum antibacterial activity and probiotic properties such as the tolerance under low pH, no hemolytic activity, and susceptibility to several antibiotics. *T. castaneum* increased its resistance against Bt infection after feeding on *E. mundtii* (Grau et al., 2017). The intestinal bacteria *E. mundtii* of *Spodoptera littoralis* produces an antimicrobial peptide mundticin KS, which can strongly inhibit some potential pathogens and weaken their colonization, which promote the stability of the intestinal microbiota against bacterial infections (Shao et al., 2017). It is worth noting that the same intestinal bacteria may play contrary roles in different insects. For example, after the reintroduction of *E. mundtii* into *P. xylostella* without intestinal microbiota, the host regained its sensitivity to Cry1Ac protoxin (Li et al., 2021).

### Other Complicated Resistance Mechanisms

The resistance mechanisms of some insects to Cry toxins are extremely complex (Patil et al., 2013; Melo et al., 2016), rendering crop pest control very difficult (Wang et al., 2018). In addition to receptors, changes in the expression levels of other proteins also seem to affect the insect resistance (Wei et al., 2018). Indeed, the mid-intestinal epithelial cell transcriptome assay of *P. xylostella* reveals that 28 chymotrypsin and 53 ABC transporters are related to insect resistances (Xie et al., 2012). Taking sensitive strain BtS and resistant strain AbR in Asian corn borer (*Ostrinia furnacalis*) as the research objects, scientists found that Cry1Ab resistance is associated with the changes in protein transcription levels involved in the insect growth regulation and metabolism after a transcriptome analysis (Xu et al., 2015).

Insect detoxification enzymes also seem to exhibit an important impact on the development of resistance mechanisms. For examples, the glutathione-S-transferase synthesized by subalpine mosquito (*Aedes rusticus*) exhibits a detoxification function to promote its resistance against Bti (Boyer et al., 2012). Another example comes from *D. virgifera* that has evolved resistance and cross-resistance to various Cry toxins in the field (Gassmann et al., 2020); indeed, the expression levels of esterase and dynein in *D. virgifera*-resistant populations are found to be upregulated, which may be involved in the processes of detoxification and mid-intestinal repair to increase resistance (Zhao et al., 2019).

The Cry toxin receptor is not evolutionally conservative to confer resistance of insects. Many Hemiptera insects, including aphids, are not sensitive to Cry toxins. These Cry toxins can be activated in the intestines of Hemiptera insects (Shao et al., 2016) but cannot interact with their potential receptors (Shao et al., 2013). Studies have shown that APN, ALP, and cadherin proteins in the mid-intestine of the soybean aphid (*Aphis glycines*) are not conserved among other insect species, so Cry toxins that are generally effective against lepidopteran insects cannot work on them (Liu et al., 2012).

The unique feeding and digestion characteristics of insects are also responsible for their resistance against Cry toxins. For example, when *A. mellifera* adults and worker larvae are fed with high concentrations of Cry1C and Cry2A, the activities of their four intestinal enzymes, BBMV structure, and survival and development are all not affected, which is thought to be related to its special feeding biology and digestion physiology. Yet, the precise mechanism remains to be determined (Wang et al., 2017). Cry1Ac can be activated in the intestine of *A. pisum* to bind to the intestinal epithelium mediated by glycan, but with very low aphicidal activity (Li et al., 2011). This may be because aphids ingest and expel a large amount of liquid food quickly, causing the activated Cry1Ac toxin to stay in the intestines far too short to exert observable damage.

## Conclusion and Outlook

Compared with the traditional chemical insecticides, Bt preparations and Bt-transgenic crops are distinguished by their high specificity and environmental safety. However, with their large-scale commercial applications, more cases of insect resistance have emerged (Bravo et al., 2011), which makes this agent as “the most successful microbial insecticide in the world” doubtful (Baragamaarachchi et al., 2019). Although a certain degree of understanding on the insecticidal mechanisms and resistance mechanisms has been achieved, it is obvious that this knowledge is far from complete. The battle between the Bt or Cry toxins and target insects is still continuing. Although the intestinal microbiota and epigenetic mechanisms mentioned above can promote the development of insect resistance, it, like a coin, has two sides. To wrestle against the host, Bt may enhance the toxicity of Cry toxins by exploiting the intestinal microbiota (Broderick et al., 2009; Paramasiva et al., 2014) or by interfering with the epigenetic mechanism of the insect host to affect the expression of immune and development-related genes (Bierne and Nielsen-LeRoux, 2017; Baradaran et al., 2019; Özbek et al., 2020).

To further improve the insecticidal efficiency of Cry toxins and to reduce the insect resistance, we propose to start from the following aspects.

First, it is necessary to speed up the search for new Bt strains to more comprehensively screen and identify new Cry toxins (Pinos et al., 2021) and to uncover more details about the insecticidal mechanism of the toxins.

Second, it is also urgent to thoroughly investigate the insect resistance mechanisms, especially on the applications of various high-throughput sequencing technologies and multiomics techniques (e.g., transcriptomic, proteomics, metabonomics, and epigenomics), to enrich the database of insecticidal proteins or rapidly screen the vital resistance genes (Dhania et al., 2019). By artificially selecting new types of resistant insects in the laboratory, one can foresee farther the possible resistant pathways of insects; in the same token, scientists can also use control strategies for insect resistance issue purposefully, such as the use of genetic engineering, synthetic biology, and other technologies (Vílchez, 2020) to carry out a directed evolution of Cry toxins by constructing various Cry mutants for enhancing its virulence, or expanding its insecticidal spectrum; or, for targeting insects, by using CRISPR/Cas9-based gene manipulation technology to restore resistant mutants back to the wild type (Esvelt et al., 2014), as well as using the mating and reproduction characteristics of insects to reduce the number of resistant populations.

Third, for practical field application, it is essential to avoid long-term exposure of insects to Bt preparations or Cry toxins; thus, one can employ the epigenetic mechanisms to evolve resistance fast. One should insist on using the “High Dose Refuge Strategy” to rationally arrange the planting patterns of Bt-transgenic crops (Bravo et al., 2011; Xiao and Wu, 2019). It is also necessary, in view of the complexity of insect resistance mechanisms (Pinos et al., 2021), especially for the insect cross-resistance, to use a variety of Bt preparations or the combination of Bt preparations and other insecticides to produce a synergistic insecticidal effect. These may allow one to significantly promote the green and sustainable development of agriculture in the future.

## Author Contributions

LL wrote the original draft. JH and S-HC revised the manuscript. JH coordinated the work. JH and JW acquired funding. All authors contributed to the article and approved the submitted version.

## Conflict of Interest

XZ was employed by Qingdao Vland Biotech Inc. The remaining authors declare that the research was conducted in the absence of any commercial or financial relationships that could be construed as a potential conflict of interest.
